# The impact of community children's playgrounds on neighborhood interactions and residents' mental health from an all-age sharing perspective

**DOI:** 10.3389/fpubh.2025.1572192

**Published:** 2025-07-15

**Authors:** Yilin Jin, Yuetong Wang, Ning Sun, Linquan Chen, Zhenhua Zheng

**Affiliations:** University of Shanghai for Science and Technology, Shanghai, China

**Keywords:** community residents, mental health, age differences, neighborhood interaction, community children's playground

## Abstract

**Background:**

The outbreak of COVID-19 and rapid urbanization in China have put mental health at the forefront of societal concerns. Despite extensive studies on how community environments influence residents' mental health, the mechanisms underlying how community children's playgrounds within community environments influence residents' mental health across different age groups remain insufficiently studied.

**Methods:**

The study is based on 1,159 questionnaire survey data from 14 districts in Shanghai in 2022. Descriptive statistics and structural equation modeling are used for the statistical analysis of the data.

**Results:**

The findings reveal variations in the mental health status of Shanghai residents across different ages, with depression rates being higher in the young and middle-aged groups than in the older adult groups. Moreover, there are differences in the influence of community children's playgrounds on the mental health of residents across different age groups. Community children's playground influences the mental health of residents in the young adult and middle-aged groups, while neighborhood interaction has a more pronounced influence on the mental health of middle aged and older adult groups. The influence of children's playgrounds on children's mental health is more realized through the intermediary role of neighborhood communication, and the influence on the mental health of the older adult is completely realized through the intermediary role of neighborhood communication.

**Conclusion:**

In future community planning, renovation, and development, it is essential not only to meet the distinct needs of various age groups but also to pay more attention to the needs of young adults to enhance their mental health and ultimately improve the mental health of all residents.

## 1 Introduction

Multiple reports have demonstrated an increase in depression and anxiety rates throughout the population spectrum since the outbreak of the COVID-19 pandemic (1–5). This rise in prevalence has generated widespread concern regarding residents‘ mental health globally. Parallel to the growth of urbanization in China, significant improvements have occurred in resource allocation, production efficiency, and quality of life (6). However, the expansion of urbanization has led to new environmental and social issues that now directly threaten residents' mental health and well-being (7).

An increasing body of research indicates that the community environment, including the built environment and social environment, is related to mental health (8, 9). The built environment includes community green spaces, public service facilities, and housing. Community green spaces, in particular, offer residents a place to exercise and socialize, which can help to alleviate mental stress, restore attention and energy, and foster physical and mental well-being (10, 11). Other research has found that the relationship between community green spaces and mental health varies depending on gender, life stage, or the type of green spaces to which individuals are exposed (12, 13).

Therefore, further exploration of the advantageous effects of community green spaces on mental health is necessary. The children's playgrounds are considered a sub-type of community green spaces and serve as convenient neighborhood spaces for children to play and interact, providing various opportunities for interaction among neighbors (14, 15). Horton and Kraftl (71) found that children's playground have different social functions in different communities. Children perceive tranquil and safe playgrounds as spaces for chatting with friends, while playgrounds with a friendly family atmosphere are mainly used for family games (16–19). Nevertheless, prior research has mainly focused on the physical and mental benefits of engaging in sports activities on playgrounds, with little attention given to the influence of community children's playground on mental health. However, non-sport-related neighborhood interactions, such as talking and dog walking, are common on or near playgrounds. Studies have shown that community green spaces can foster positive neighborhood interaction among residents, which is advantageous for overall health and well-being, social integration, community development, and social cohesion (20).

The social environment primarily encompasses aspects such as neighboring interaction, community safety, and social networks. Among them, neighboring interaction is crucial in sustaining social and physical activities, thus leading to a healthier mental state. The underlying mechanism has three aspects: dissemination of information regarding mental health, enhancing the mental health of residents by involving them in social groups/activities, and enabling residents to access material and emotional support through social networks, leading to an improved mental state (21). Neighboring interaction has also been found to enhance community (22–24) and improve quality of life (25, 26). Interaction with neighbors, such as sharing personal interests or exchanging greetings, can increase happiness, health, and well-being (27). For example, Kim et al. found that interaction between people with weaker social relationships with neighbors was related to better mental health (28, 29). Casual interaction in the community makes residents feel that they are participating in their neighbors' lives, which benefits their mental health. The positive relationship between neighboring interaction and community green spaces has been widely accepted (30, 31), and the role of neighboring interaction in affecting mental health in the community environment has also been recognized by scholars. However, inadequate research has focused on the role of community children's playground in enhancing neighboring interaction and its role as a mediator in the effect of community children's playground on mental health. Therefore, studying the relationship between community children's playground in the community and neighboring interactions and their impact on residents' mental health has become increasingly necessary.

Individual factors, such as age, can influence the frequency of residents' use of community green spaces. This, in turn, can affect their satisfaction with the quality of the community environment. It can also affect neighborhood interaction, subsequently influencing their mental health (32, 70). Research has also shown that community green spaces play an important role in providing a gathering place for neighborhood interaction and children's play (33). Children prefer green spaces close to their homes, allowing them to feel comfortable and safe in familiar surroundings with their friends (34). Young people who report lower satisfaction with the community environment are more likely to report symptoms of depression (35). Young and middle-aged adults with children often use playgrounds with their children, but they tend to interact with their adult neighbors instead of engaging in play activities (36). Meanwhile, older adults may spend more time in the community and interact more with each other in the nearby green spaces than other age groups (37, 38). However, most studies have not differentiated personal factors among community residents, and few have explored differences among age groups. Therefore, our study focuses on the relationships among community children's playground, neighborhood interaction, and the mental health of Chinese residents and examines the potential associations with mental health across age groups.

Our study addresses the following research questions:

1. What is the status of Chinese residents' mental health, community children's playground, and neighborhood interaction in the community?
2. Do community children's playground significantly influence residents' mental health and neighborhood interaction? Is neighborhood interaction a mediating variable for the relationship between community children's playground and residents' mental health?
3. Are there age differences in the effects of community children's playground and neighborhood interaction on residents' mental health?

## 2 Methods and measures

### 2.1 Study population

**Figure 1:**
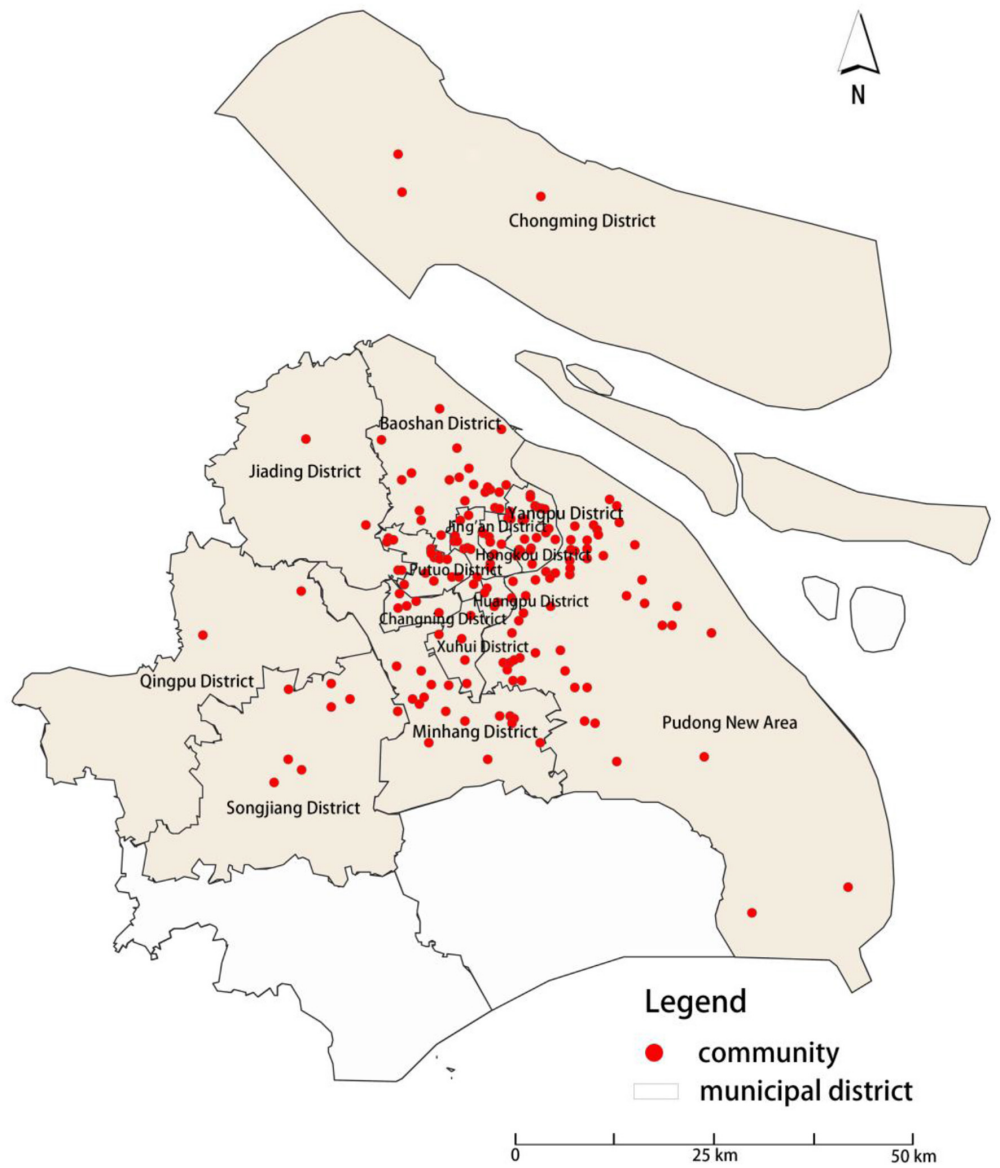
Sample sampling chart.

This study is based on survey data collected from a sample of 1,159 residents in communities in Shanghai, China. The purpose was to explore the relationship between neighboring interactions and mental health among community residents. The survey began on October 1, 2021 and ended on January 30, 2022. This survey takes diversity as the sampling principle. In order to ensure the representativeness of the samples, the community samples are selected to fully cover the communities with different geographical locations, convenient transportation, construction years and quality in Shanghai. In view of the fact that Jinshan District and Fengxian District belong to the industrial-dominated areas in the outer suburbs, and the communities are mainly populated by industrial workers, which is significantly different from the characteristics of ‘urban communities' focused in this study, the inclusion of samples may lead to bias; in addition, due to the remote geographical location of the two places and the limitation of research time and funds, it is difficult to obtain sufficient and representative samples at the same cost, so they are not included in the research scope. Finally, the survey sample covers 205 residential communities in 14 districts of Shanghai (see Figure 1 for details), covering central urban areas, suburbs and some outer suburbs, and fully presents the continuous pedigree of urban community environment, which effectively guarantees the comparability and effectiveness of the data. Under the supervision of the academic committee at Shanghai Polytechnic University, all respondents voluntarily completed the survey. To ensure the validity of the data, the survey website and login certificate were password-protected, and no access was granted without a password. In the end, 1,159 valid samples were obtained, and the samples were divided into four age groups: children (0–17 years old), young adults (18–39 years old), middle-aged adults (40–59 years old), and older adults (60 years old and older). The survey included 328 children, 251 young adults, 274 middle-aged adults, and 306 older adults. Community residents were asked to provide specific information on evaluating children's play area environments, mental health, and neighboring interactions. See Table 1 for sample statistics.

**Table 1:** The sample demographics.

**Demographics**	***N***	**%**
**Age**		
Children	328	28.30
Young adults	251	21.66
Middle-aged adults	274	23.64
Older adults	306	26.40

### 2.2 Measurement

#### 2.2.1 Dependent variable: mental health

This study assesses the mental health status of individuals across various age groups through self-assessment. The WHO-5 has demonstrated high reliability and validity in multiple mental health studies in different countries and populations (39, 40), and the Chinese version of the WHO-5 has shown strong consistency in practical research applications within the field of public health in China. The Chinese version of the WHO-5 evaluates depression by assessing five positive emotions experienced in the past two weeks: (1) feeling happy and comfortable; (2) feeling calm and relaxed; (3) feeling full of energy; (4) feeling clear and well-rested after waking up; and (5) feeling that daily life is filled with interesting things. The mental health status of residents was assessed by the frequency of being asked about these five positive emotions in the past 2 weeks. Each item adopts a 6-level scoring system (0–5 points), 0 = no; 1 = a small part of time; 2 = less than half of the time; 3 = more than half of the time; 4 = most of the time; 5 = always, the total score range is 0–25 points. This paper measures the mental health status of people of different ages. A total score of <13 points indicates depression. The higher the score, the better the mental health status.

#### 2.2.2 Independent variable: community children's playground

**Figure 2:**
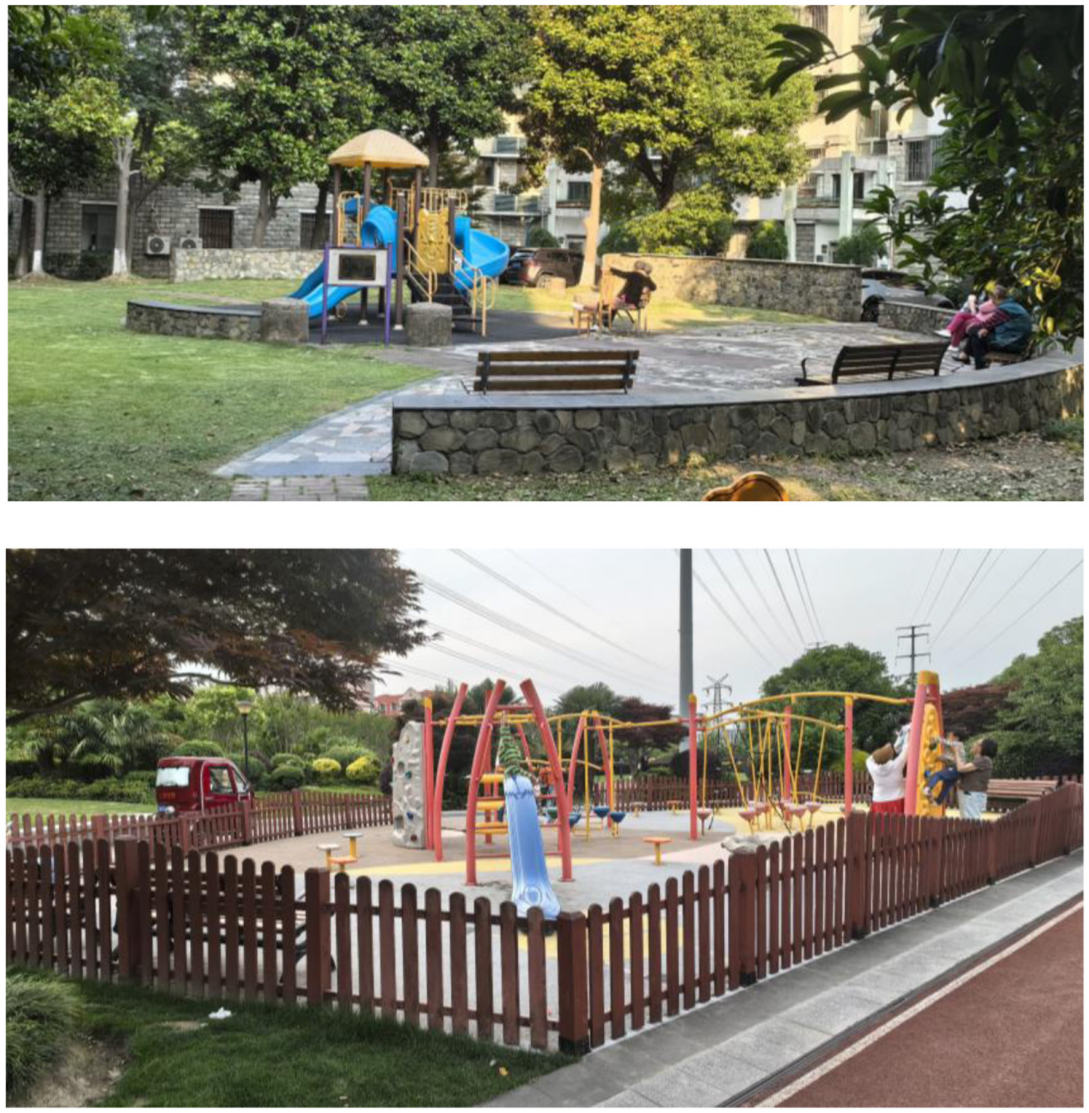
Typical community children's playground environment example.

In the community, children's play areas are a part of community green spaces (41). This study defines the children's playground as the designated space for children's play within the community. The environment of the community children's playground encompasses five factors: “the provision of convenient seating areas in the play areas within the community,” “the community provides many facilities for children's play,” “I often take my child to play in the children's play area in the community,” “the community provides many spaces for children's play,” and each response is scored from 1 to 5, where 1 represents “strongly disagree,” 2 represents “disagree,” 3 represents “neutral,” 4 represents “agree,” and 5 represents “strongly agree.” Higher scores indicate a higher acceptance of the community's play areas. If a respondent's community lacks a designated children's play area, their score will be assigned a value of 0. Figure 2 shows the environmental conditions of children's playgrounds (including rest seats and recreational facilities) in two communities in Shanghai as an example of different types of venues.

#### 2.2.3 Mediating variable: neighborhood interactions

The quality of neighborhood interaction can be measured by investigating how many people in an individual's social network are based on weak or strong social ties (42, 43). In our study, the value of neighborhood interaction was measured by residents' subjective evaluations of the importance of neighborhood interaction. Neighborhood interaction was measured in five areas: the mutual help between you and the people in your community (such as taking care of children, buying things for each other, and borrowing tools from each other), the consultation and communication of personal affairs between you and the people in your community (such as child-rearing and fitness exercises), gathering or participating in collective activities with the people in your community, and communicating at home or on the street with the people in your community. Each item was scored from 1 to 4 (1 = never, 2 = rarely, 3 = sometimes, 4 = often) to represent the frequency of neighborhood interaction.

#### 2.2.4 Control variables

In this study, education level, age, gender and per capita disposable income (ten thousand yuan) at the district level were included in the conceptual model as control variables. Education level is categorized based on the following values: 1 for those who completed primary school or less, 2 for those who finished high school, vocational school, or technical school, 3 for those who hold a college diploma, 4 for those who earned a bachelor's degree, and 5 for those who attained a master's degree or more. Gender is assigned a value of 1 for males and 2 for females.

### 2.3 Statistical analysis

This study utilized descriptive statistical analysis and structural equation modeling (SEM) to explore the complex relationships between community children's playground, neighborhood interaction, residents' mental health, and differences among different age groups. SEM is advantageous in quantitative research involving multivariate interaction and inter-group comparisons. This study had a sample size of 1,159 (>1,000), with sample means that were approximately normally distributed. The asymptotically distribution-free (ADF) statistical method was chosen since it is appropriate for SEM analysis.

To validate the data for SEM analysis, we conducted a multi-factor confirmatory analysis of the measurement models in the conceptual model. The composite reliability of the three measurement models of community children's playground, neighborhood interaction, and residents' mental health were 0.993, 0.916, and 0.951, respectively, exceeding the standard of 0.7. The average variance extracted (AVE) of the measurement models were 0.973, 0.731, and 0.794, respectively, exceeding the standard of 0.5 (44). The standardized factor loading and reliability coefficient (SMC) of the observed variables are greater than the standards of 0.6 and 0.36, which have been widely used (45, 46). See Table 2 for details. All measurement models have good reliability and validity, making them suitable for structural equation modeling analysis. The model fitting results also indicated that the fit indices CFI > 0.90, AGFI > 0.90, TLI > 0.90, and RMSER <0.08 all meet the ideal standard, indicating that the model fits well.

**Table 2:** Validity and reliability test results of variables.

**Measurement model (CFA)**	**Observed variables**	**Estimates of model parameters**				**Convergent validity**			
		**Non-standardized factor load**	**Standard error (SE)**	**Composite reliability (CR)** ***t*****-value**	***p***	**Standardized factor load**	**Reliability coefficient SMC**	**Composite reliability (CR)**	**Average variance extracted (AVE)**
Mental health	Depre 1	0.929	0.022	42.800	^***^	0.900	0.810	0.951	0.794
	Depre 2	0.952	0.023	41.117	^***^	0.893	0.797		
	Depre 3	1.013	0.022	46.320	^***^	0.915	0.837		
	Depre 4	0.943	0.024	38.771	^***^	0.868	0.753		
	Depre 5	1				0.879	0.773		
Children's playground	CHP_seat	1.012	0.007	140.152	^***^	0.979	0.958	0.993	0.973
	CHP_instr	1.004	0.005	195.452	^***^	0.992	0.984		
	CHP_chil	1.001	0.007	144.655	^***^	0.980	0.960		
	CHP_spa	1				0.994	0.988		
Neighborhood interaction	lnter_1	1				0.806	0.650	0.916	0.731
	lnter_3	1.109	0.032	34.300	^***^	0.882	0.778		
	lnter_4	1.164	0.035	33.424	^***^	0.867	0.752		
	lnter_5	1.097	0.035	31.549	^***^	0.863	0.745		

^***^ means significant at the 1% confidence level.

## 3 Results

### 3.1 Descriptive statistics

Tables 3, 4 present descriptive statistics indicating that 14.32% of the participants scored <13 points on the Personal Health Assessment, suggesting that 14.32% of the residents suffered from depression, revealing some mental health issues among Chinese residents. The depression rate for individuals in the youth group was significantly higher than that for both the children's and middle-aged groups, with a rate of 19.92%. In contrast, the older adult group recorded a lower depression rate of 10.78%. Moreover, for all observed variables related to mental health and neighboring socialization, the mean values of the children's and older adult groups were higher than those of the youth and middle-aged groups. The mean score for community children's playground was between 1 and 3, indicating poor environmental conditions. In the control variables, the average education level of residents is high school, secondary school and technical school, the average age is 38.67 years old, and the gender ratio gap is not large. The average disposable income of residents is 6.81 ten thousand yuan.

**Table 3:** Variable descriptive statistics.

**Latent variables**	**Observed variables**	**Variable items**	**Mean scores**				
			**All**	**Children**	**Young adults**	**Middle-aged adults**	**Older adults**
Mental health	Depre_1	Feeling happy and comfortable	4.59	4.71	4.56	4.47	4.57
	Depre_2	Feeling calm and relaxed	4.45	4.59	4.26	4.38	4.54
	Depre_3	Feeling energetic	4.37	4.65	4.25	4.26	4.26
	Depre_4	Feeling awake when you wake up and getting enough rest	4.39	4.45	4.13	4.37	4.54
	Depre_5	Everyday life is full of interesting things	4.32	4.56	4.23	4.16	4.28
Children's playground	CHP_seat	The provision of seating areas in the play areas within the community, which is extremely convenient	2.01	2.34	2.24	1.84	1.63
	CHP_instr	The community provides many facilities for children's play	1.95	2.27	2.16	1.76	1.63
	CHP_chil	I often take my child to play in the children's play area in the community	1.96	2.27	2.17	1.72	1.66
	CHP_spa	The community provides many spaces for children's play	1.94	2.26	2.18	1.74	1.59
Neighborhood interaction	lnter_1	Do you and your neighbors help each other (babysitting, shopping, borrowing gadgets)	2.57	2.72	2.48	2.53	2.51
	lnter_3	You confide in your neighbors about personal matters (child rearing, fitness exercise, etc.)	2.5	2.64	2.38	2.41	2.53
	lnter_4	You get together with other people in your community or participate in group activities	2.34	2.54	2.26	2.2	2.33
	lnter_5	You and other people in your community come to your home or interact on the street	2.53	2.65	2.36	2.38	2.67
Control variable	Income	per capita disposable income (ten thousand yuan)	6.81	7.00	6.87	6.27	7.03
	Educate	What is your level of education	2.41	1.89	3.3	2.65	2.02
	Age	What is your age	38.67	13.02	24.22	49.43	68.40
	Gender	What is your gender	1.64	1.58	1.63	1.74	1.61

**Table 4:** The comparison of depression prevalence among different groups.

**Age**	***N***	**Depression percentage %**
Children	328	12.5%
Young adults	251	19.92%
Middle-aged adults	274	15.33%
Older adults	306	10.78%
Total	1,159	14.32%

### 3.2 Analysis based on the models of full sample

**Figure 3:**
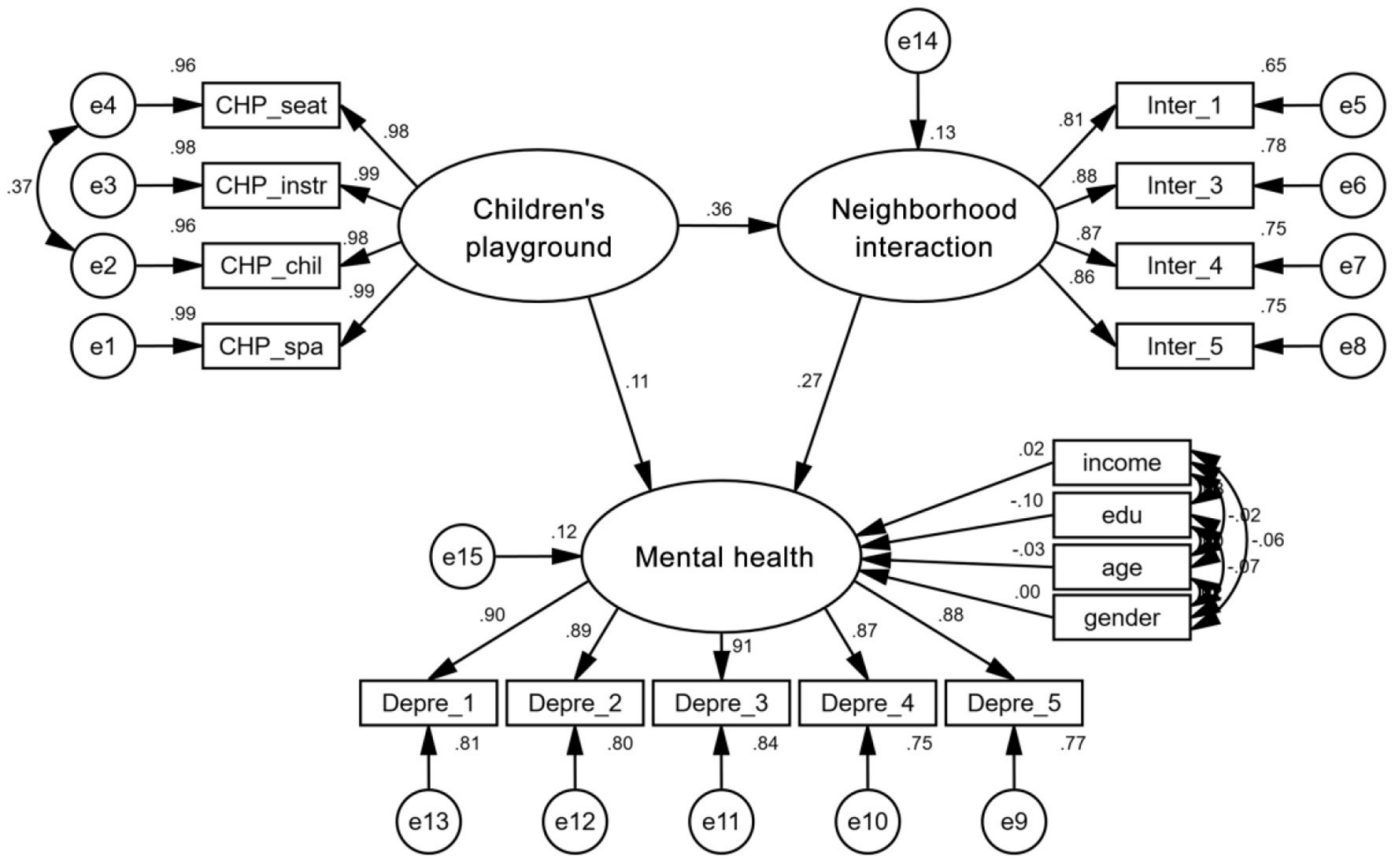
Standardized path diagram for the whole sample model.

Table 5 and Figure 3 illustrate the simulation results based on the overall sample. Regarding the total effect, after controlling for education level and housing size, children's playground and neighborhood interaction had significant positive effects on mental health, with total effect values of 0.212 and 0.273, respectively. The direct and indirect effects of the community children's playground on mental health were both significant, indicating the existence of partial intermediate variables in the pathway. The mediation effect value of neighborhood interaction was 0.099, suggesting that the positive influence of community children's playground on mental health requires improving neighborhood interaction as a means of implementation.

**Table 5:** Total, direct, and indirect effects of the full sample model.

**Independent variable**	**Intermediate variable**	**Dependent variable**		
	**Neighborhood interaction**	**Mental health**		
		**Total effect**	**Direct effect**	**Indirect effect**
Children's playground	0.362^**^	0.212^**^	0.113^***^	0.099^**^
Neighborhood interaction	–	0.273^**^	0.273^**^	–

^***^means significant at the 1% confidence level; ^**^means significant at the 5% confidence level.

The propensity score matching (PSM) was used to analyze the robustness and sensitivity of the results of the structural equation model. The sample was divided into treated and control groups based on whether the community provided many children's playground. The experimental group included 50% of the samples with the most playgrounds, while the rest were grouped into the control group. Education level, age, gender and per capita disposable income at the district level are used as interference factors through binary Logit. The model estimated the probability of each sample entering the experimental group and obtained its propensity score. Then, matching was performed by sequentially matching samples with the closest propensity scores belonging to different groups.

The radius matching method is used for matching, and the results show that the matching success rate is 100%. The propensity score matching (PSM) effect was tested by parallel hypothesis test. The results show that (Table 6), the absolute value of the standardized deviation after matching is <20 %, and the standardized deviation value decreases significantly, and the matching effect is better; the *t*-test after matching was not significant (*p* > 0.05), indicating that the matching effect was good.

**Table 6:** PSM parallel hypothesis test.

**Disturbing variable**		**Treated (experimental group)**	**Control (control group)**	**Standardized deviation (%)**	**Standardized deviation reduction (%)**	***t***	***p***
Income	Unmatched	6.868	7.226	−16.01%	−21.53%	−0.955	0.345
	Matched	6.868	7.301	−19.46%		−1.151	0.256
Education level	Unmatched	2.464	2.61	−11.58%	−28.08%	−0.687	0.496
	Matched	2.464	2.65	−14.83%		−0.872	0.388
Age	Unmatched	35.951	37.366	−6.39%	−47.07%	−0.407	0.686
	Matched	35.951	38.025	−9.40%		−0.593	0.556
Gender	Unmatched	1.626	1.634	−1.75%	−188.16%	−0.108	0.915
	Matched	1.626	1.65	−5.03%		−0.308	0.760

The average treatment effects on treated (ATT) effect analysis was performed on the matched data. The ATT effect refers to the average degree of influence of a particular treatment on the treated object, which is used to measure the causal relationship (47). According to Table 7, it can be seen that there is a difference between the number of community children's playgrounds before matching and the interesting things in daily life (*p* < 0.05). Subsequently, after matching, the ATT effect value demonstrated significance (*p* < 0.05). The PSM analysis showed a significant difference between the number of community children's playground and their well-being. The ATT effect value is 0.784, which implies a positive effect of the number of community children's playground on their well-being.

**Table 7:** Analysis of average treatment effect.

**Grouping**	**Treated (experimental group)**	**Control (control group)**	**Difference (ATT effect value)**	**Std. error**	***t***	***p***
Unmatched	4.566	3.902	0.663	0.220	3.013	0.003
ATT	4.566	3.782	0.784	0.083	9.409	0.000

This study examines whether there is a mediating effect of neighboring interaction on the influence of community children's playground on mental health. Bootstrap's bias-corrected confidence interval (CI) method, percentile CI method, and *Z* value criterion were used to ensure the rigor of the study. The total and indirect effects of the two tests do not include 0 in the lower to upper values of the 95% CI in the pathway of the influence of community children's playground on mental health, and the *Z* value is >1.96. This indicates the presence of a mediating effect, namely, that neighboring interaction has a mediating effect on the pathway of community children's playground on mental health. The lower to upper values of the direct effect include 0, and the *Z* value is >1.96, indicating that the mediator is a partial mediator. community children's playground positively influence residents' mental health through neighboring interactions as a partial mediator. The statistical results of the mediating effect test are shown in detail in Table 8.

**Table 8:** Testing the mediating effect of neighborhood interaction on the impact of community children's playground on mental health.

**Disturbing variable**	**Point estimate value**	**Product of coefficients**		**Bootstrap**			
				**Bias-corrected 95% CI**		**Percentile 95% CI**	
		**SE**	***Z***	**Lower**	**Upper**	**Lower**	**Upper**
Children's playground → Mental health	**Total effects**						
	0.136	0.018	7.556	0.106	0.168	0.109	0.170
	**Direct effect**						
	0.072	0.017	4.235	0.044	0.103	0.044	0.101
	**Indirect effects**						
	0.063	0.010	6.300	0.048	0.078	0.049	0.083

### 3.3 Comparison of model differences among different age groups

**Figure 4:**
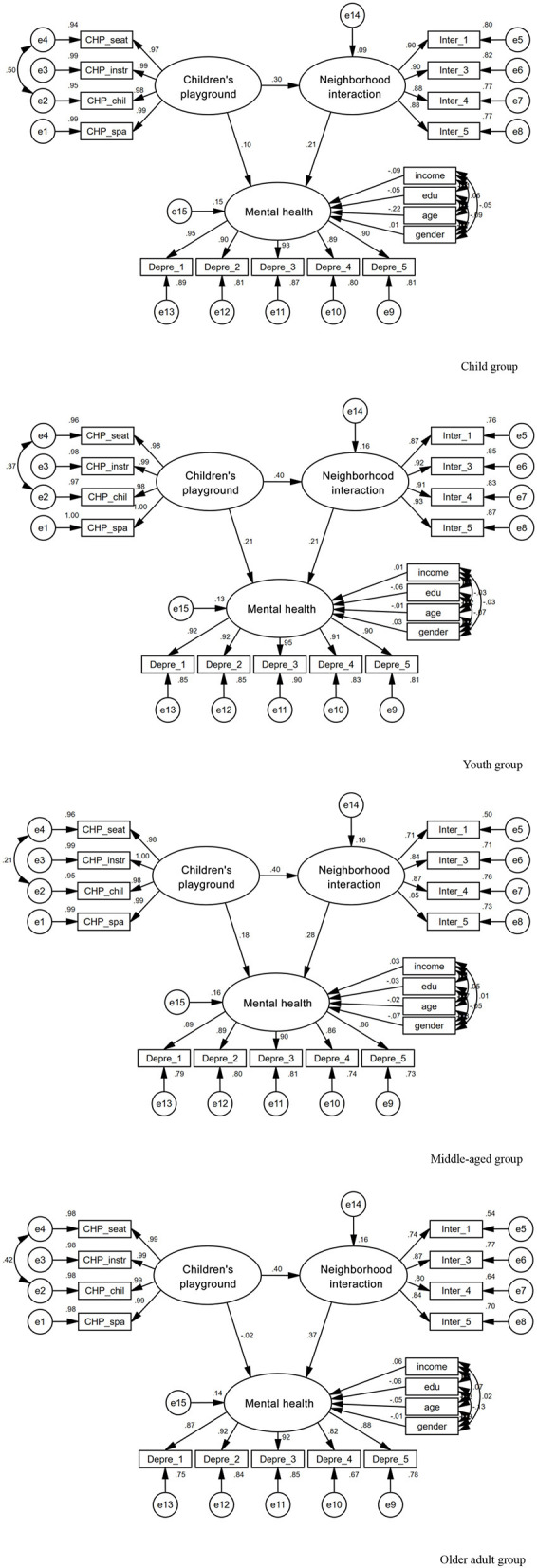
Comparison of standardized path diagrams for different age groups.

We compared the model paths for different age groups. We set the output result coefficient to a consistent *p* value of < 0.05, indicating statistically significant differences in the model paths for different age groups (Table 9, Figure 4). Children's mental health was positively affected by community children's playgrounds and neighborhood interactions, and the total effect values were 0.166 and 0.215, respectively. The direct and indirect effects of community children's playgrounds on children's mental health are significant, indicating that there is a partial mediating effect in the path, and the mediating effect value of neighborhood interaction is 0.065. This shows that the positive impact of community children's playgrounds on children's mental health needs to be achieved by improving neighborhood interaction.

**Table 9:** Comparison of the model paths in different age groups.

**Independent variable**		**Intermediate variable**	**Dependent variable**		
		**Neighborhood interaction**	**Mental health**		
			**Total effect**	**Direct effect**	**Indirect effect**
Children	Children's playground	0.305^**^	0.166^***^	0.100^*^	0.065^***^
	Neighborhood interaction	–	0.215^**^	0.215^**^	–
Young adults	Children's playground	0.397^**^	0.298^**^	0.214^**^	0.084^***^
	Neighborhood interaction	–	0.212^***^	0.212^***^	–
Middle-aged adults	Children's playground	0.402^**^	0.290^**^	0.178^**^	0.112^**^
	Neighborhood interaction	–	0.279^***^	0.279^***^	–
Older adults	Children's playground	0.396^**^	0.123^*^	−0.025	0.148^***^
	Neighborhood interaction	–	0.374^***^	0.374^***^	–

^***^means significant at the 1% confidence level; ^**^means significant at the 5% confidence level; ^*^means significant at the 10% confidence level.

Young adults' mental health is positively influenced by their access to playgrounds and neighborhood interaction, with total effect values of 0.298 and 0.212, respectively. The direct and indirect effects of playgrounds on young adults' mental health are significant, indicating a partial mediation effect. The mediation effect of neighborhood interaction is 0.084, suggesting that increasing neighborhood interaction is necessary to achieve the positive influence of community children's playground on young adults' mental health.

Middle-aged adults' mental health is positively influenced by their access to playgrounds and neighborhood interaction, with total effect values of 0.290 and 0.279, respectively. Playgrounds' direct and indirect effects on middle-aged adults' mental health are significant, indicating a partial mediation effect. The mediation effect of neighborhood interaction is 0.112, suggesting that increasing neighborhood interaction is necessary to achieve the positive influence of community children's playground on middle-aged adults' mental health.

Older adults' mental health is positively influenced by their access to playgrounds and neighborhood interaction, with total effect values of 0.123 and 0.374, respectively. The indirect effect of playgrounds on older adults' mental health is significant, while the direct effect is not significant, indicating the presence of a complete mediation effect. The mediation effect of neighborhood interaction is 0.148, indicating that the positive influence of community children's playground on older adults' mental health is achieved entirely through enhancing neighborhood interaction.

## 4 Discussion

Our research explored the complex relationships among community children's playground, neighborhood interaction, residents' mental health, and the differences across age groups. Our study confirmed the variance in mental health conditions among other age groups; specifically, youth groups showed higher depression rates than children and middle-aged adults, whereas older adult groups showed lower depression rates, corroborating some previous studies (48–50).

Our study additionally confirmed the significant influence of community children's playground and neighborhood interaction on residents' mental health. Moreover, our findings indicated that community children's playground contribute significantly to neighborhood interaction, consistent with some previous research (51). More specifically, our study verified that neighborhood interaction has a more significant effect on residents' mental health than community children's playground, highlighting the crucial role of neighborhood interaction in residents' mental health, which aligns with some previous studies (52–54).

The relationship between community children's playground, neighborhood interaction, and residents' mental health varies significantly across age groups. Our study found that older adult residents enjoy better mental health conditions than other age groups. In contrast, young adults have a higher risk of depression than other age groups. One explanation for this phenomenon could be that aging has a positive effect, as previous research has shown that aging is linked to a preference for positive memories, including pictures, faces, and vocabulary (55), which may contribute to good mental health among older adult residents. In contrast, young adults are more likely to engage in competitive environments in pursuit of academic or career achievement, which may lead to fear of failure (56), increasing the risk of depression for young adults compared to other age groups.

The influence of children's play areas on the mental health of young and middle-aged adults is more significant than that of children and older adults. One possible explanation for this phenomenon is that young and middle-aged individuals experience greater social and work-related stress than children and older adults, requiring a place to relax and relieve stress. Williams (72) has suggested that neighborhood interaction is stimulated when residents can come into contact, live close to each other, and have appropriate spaces for socializing (43). Children's play areas can provide young and middle-aged adults with a nearby social venue and promote informal neighborhood interaction and social behavior, thereby improving their physical and mental health. The reasons underlying the significant influence of children's play areas on the mental health of young adults may be relatively complex. Research indicates that the perceived neighborhood environment has an indirect positive influence on mental health, with healthy practices acting as mediators in the relationship between the perceived neighborhood environment and mental health, and the influence of healthy practices on the mental health of young people is significantly greater than that of older adults (57). As such, healthy behavior can be viewed as a mediator that explains the greater influence of children's play areas on young people's mental health than on older adults. Further, Guite et al. (73) found that residents' dissatisfaction with social and recreational facilities lowers their level of mental health based on a large-scale survey of Greenwich in London. Middle- aged parents prioritize the safety and hygiene of children's play areas, whereas children prefer to enjoy games and adventures (58, 59). Veitch (65) also found that children are not concerned with elaborate equipment or venues as long as they have a safe place to play with their friends, which could be a reason why middle-aged adults' mental health is more affected by children's play areas than that of children. For older adults, the perception of the physical environment of the community may be influenced more by emotional needs; some argue that as they become aware of the shortening of their remaining lifespan, they will prioritize emotional goals, which may explain why the influence of children's play areas on the mental health of older adults is likely to be smaller than that on other age groups. There is little age difference in the influence of children's play areas on neighborhood interaction.

Our study found that neighborhood interaction has a greater influence on the mental health of middle-aged and older adults than on children and young adults. One possible reason is the effect of personal factors such as age and employment status. Age can influence the frequency with which residents use community facilities, which can, in turn, affect neighborhood interaction (32). Additionally, middle-aged and older adults may have lived in the community longer than children and young adults (60, 61). Length of residence has been found to be a positive indicator of neighborhood interaction among neighbors, and an increase in social networks occurs throughout one's life (62). As social partners are eliminated from one's social network, middle-aged and older adults tend to spend more time with intimate network members such as family and neighbors, thus increasing the depth of neighborhood interaction. As such, neighborhood interaction may have a greater influence on the mental health of middle-aged and older adults than on children and young adults. Another possible reason is the difference in life experiences, needs, and expectations between middle-aged and older adults and children and young adults. When the future is perceived as unlimited, individuals prioritize optimizing future goals, while the maximal pursuit of current emotional goals is driven by the perception of limited future time (63). Therefore, children and young adults may focus on self-growth and future exploration. In contrast, middle-aged and older adults may focus more on strengthening positive states and emphasizing emotional exchanges. Emotional reactivity also increases with age (64). Thus, middle-aged and older adults may require more neighborhood interaction to increase emotional support and mental health.

In the children group and the older adult group, our study found that the impact of community children's playgrounds on children's mental health is more achieved through the intermediary role of neighborhood interaction, and the impact on the mental health of the older adult is completely achieved through the intermediary role of neighborhood interaction. This shows that community children's playgrounds play an important role in the mental health of children and the older adult. A feasible explanation is that the children's favorite places are usually near their homes because these places are familiar and shared with friends, and this gives children a sense of comfort and safety (34). In addition, children strongly desire neighborhood interaction, and the presence of friends is often an important factor for children's outdoor games (65). Therefore, community children's playground provide a familiar space close to home that satisfies their neighborhood interaction needs. In addition, children can establish closer social connections in community children's playgrounds, so as to enhance their mental health. Perhaps this can explain that the impact of community children's playgrounds on children's mental health is more achieved through the intermediary role of neighborhood interaction. Older adults can increase their psychological influence by entering community children's playground and engaging in neighborhood interaction. This may be because playgrounds facilitate inter-generational use, with potential physical and mental health benefits for older adults. The site facilities can attract a wider range of users to engage in different activities (such as exercise and play), cultivating community awareness, promoting neighborhood interaction, and developing inter-generational relationships (66, 67). Effective participation in neighborhood and inter-generational interactions can help older adults maintain good mental health and ideal cognition (68).

The community children's playground have different influences on the mental health of residents of various ages. To improve residents' mental health, it is necessary to consider the needs of distinct age groups and propose targeted recommendations and strategies based on their characteristics. First, community children's playground have a positive effect on residents' mental health. Improvements to these playgrounds should take a multifaceted approach, such as renovating the facilities, increasing natural contact, and adding more recreational equipment. Youth and middle-aged adults have higher rates of depression than children and older adults and community children's playground have a greater impact on their mental health. Therefore, improving the playground's environment to a certain extent can enhance young and middle-aged residents' mental health. Children and older adults are more vulnerable groups and tend to have more limitations on their activities than adults (69). They are also groups of the utmost importance in the community, as their mental health is more affected by neighborhood interactions, which, in turn, are influenced by community children's playground. Therefore, in building community children's playground, decision-makers and environmental designers should pay particular attention to the needs of children and older adults regarding neighborhood interaction. For instance, renovating seating in community children's playground to accommodate older adults will allow them to participate in inter-generational activity or neighborhood interaction more comfortably. The seating design should provide an adequate backrest and handrails to offer support and security. Furthermore, adding equipment that appeals to various age groups, interests, and abilities can help promote interaction and enhance social skills among children. In summary, improving children's and older adults' approval of community children's playground is crucial to address their mental health problems effectively.

This study has several limitations. First, the survey scope and sample size are limited. The research findings cannot represent all community environments in China, and more empirical research needs to be conducted. Second, the representative of the resident sample needs to be further improved. Finally, since the community children's playground in this study are based on subjective evaluations, our subjective assumption of population distribution is static and does not consider human mobility. Follow-up studies should combine subjective and objective environmental evaluation systems and use more objective data to better explore the relationship between community children's playground and residents' mental health.

## 5 Conclusion

Our results show that there are differences in the mental health status of Chinese residents. Compared with the depression rate of children and middle-aged groups, the depression rate of the young group is higher, while the depression rate of the older adult group is lower. Therefore, special attention should be paid to the mental health problems of young people. Our study also confirms that community children's playgrounds have a significant impact on residents' mental health. At the same time, neighborhood interaction is a mediating variable for community children's playgrounds to affect residents' mental health. More importantly, we found that community children's playgrounds have different effects on the mental health of residents of different ages. Community children's playgrounds have a greater impact on the mental health of young and middle-aged groups. There is a small age difference in the impact of community children's playgrounds on neighborhood interaction. Neighborhood interaction has a greater impact on the mental health of middle-aged and older adult groups. The impact of community children's playgrounds on children's mental health is more achieved through the intermediary role of neighborhood interaction. The impact of community children's playgrounds on the mental health of the older adult is entirely achieved through the intermediary role of neighborhood interaction. The conclusions of this paper provide new ideas for the construction, improvement and renewal of community children's playgrounds, and help policy makers and designers to re-examine the positioning of end users to promote more health protection.

## Data Availability

The datasets presented in this article are not readily available because they contain information that could compromise the privacy of research participants. Requests to access the datasets should be directed to zhenhuazheng@usst.edu.cn.
